# The importance of reaction time to the starting signal on race results in elite motorcycle speedway racing

**DOI:** 10.1371/journal.pone.0281138

**Published:** 2023-01-27

**Authors:** Maciej Markowski, Stefan Szczepan, Marek Zatoń, Sarah Martin, Kamil Michalik

**Affiliations:** 1 Faculty of Physical Education and Sport, Wroclaw University of Health and Sport Sciences, Wroclaw, Poland; 2 Department of Swimming, Faculty of Physical Education and Sport, Wroclaw University of Health and Sport Sciences, Wroclaw, Poland; 3 Department of Physiology and Biochemistry, Faculty of Physical Education and Sport, Wroclaw University of Health and Sport Sciences, Wroclaw, Poland; 4 School of Sport, Exercise and Rehabilitation, University of St Mark and St John, Plymouth, Devon, United Kingdom; 5 Department of Human Motor Skills, Faculty of Physical Education and Sports, Wroclaw University of Health and Sport Sciences, Wroclaw, Poland; Qatar University, QATAR

## Abstract

The study aimed to determine whether the reaction time (RT) to the starting signal has an impact on the points scored by elite male motorcycle speedway riders, or whether it depends on the starting position (gate). Differences among junior and senior riders, and how it changes during a single match (15 heats) and in the subsequent phases of the competitive season (the main and knockout phases) were investigated. The database of reaction times to the starting signal obtained by motorcycle speedway riders was collected from a mobile application called PGE Ekstraliga ver. 1.0.66 (PGE Ekstraliga, Warsaw, Poland). The database included 1.261 results obtained by 65 male riders (age 25.9 ±7.6 years), competing in the highest league in Poland (PGE Speedway Ekstraliga) in the 2021 competitive season. Reaction time was measured using the Pegasus Speedway © telemetry system (Black Burst, Warsaw, PL). Riders scoring 3 points during a heat had the fastest reaction time (F_(3,1257)_ = 8.90, p<0.001, η^2^ = 0.02), but RT did not influence the final result of the match (p<0.130). The times differ depending on the occupied starting position (F_(3,1257)_ = 6.89, p<0.001, η^2^ = 0.02), with the fastest RT in the inner position–A compared to the B (p<0.05) and C (p <0.001) positions. Senior riders showed significantly faster RT (0.246s) compared to junior ones (0.258s) (p<0.001). The width of the starting line affects the reaction time (F_(3,1257)_ = 7.94, p<0.001, η^2^ = 0.02). In the last (15^th^) heat of the match, RT was the fastest. The fast reaction time during the start affects the scoring of more points in a heat but depends on riders’ experience, the starting position and the straight width of the motorcycle speedway stadium. Coaches should pay attention to these factors when programming training measures.

## Introduction

The Polish speedway competition league (PGE Speedway Ekstraliga) is considered the best in the world and attracts outstanding riders from many countries. Motorcycle speedway riders can be classified according to the age category of junior riders (youth riders) ≤21 years of age and senior riders. No restrictions in league competitions on motorcycles with a maximum capacity of 500 cm3 (no gearbox, one gear, no brakes) apply after the age of 16 years. During a heat, four riders cover four laps of the track (length: approx. 260–425 m) counterclockwise, which takes approximately 60 seconds [1]. The starting positions (gates) A, B, C, and D indicate the place on the track from which a rider starts a heat. A is the position closest to the inner side of the track, and D is closer to the board (air-fence) that surrounds the track [2]. The start/finish line, depending on the track, are 10–12 meters wide. All speedway heats have a stationary start and during a heat, on the starting signal (lifting the start tape), riders move as quickly as possible and accelerate to a speed of over 100 kilometres per hour, reaching 80 kilometres per hour in about 2.4 seconds [1]. Traditional scoring gives 3 points for winning a single heat, 2 points for 2nd place, 1 point for 3rd place and no points for the last place [3]. A speedway match in the Polish league consists of 15 heats, with the speedway competitive season consisting of the main (match and rematch) and knockout rounds (semi-finals and finals). Due to the limited opportunity for overtaking, starting acceleration has a significant importance on final position [4] which justifies the need for further research into the starting phase of a motorcycle speedway race and the impact on the results achieved by riders.

In the past, only a few aspects of motorcycle speedway racing were investigated. The importance of the starting acceleration and reaching the maximum speed was determined [5], and it was established that there is no evidence of a relationship between the heart rate and the finishing position during a heat [4]. The relationship between the starting position, the place on the first corner and the final result in a heat were also investigated. Doggart et al. [2] suggest that starting gate position is not as influential on final race position when compared to the position of the rider at the first corner. Both are significantly correlated to final race position, however the correlation statistic suggests the stronger relationship (r = 0.42 vs r = 0.24) in favour of the first corner position. The starting position was also analysed in the context of scoring points in the motorcycle speedway world championship [6]. Interestingly, no studies have determined the importance of a rider’s reaction time and skill during the beginning of a heat. These may be factors that will help riders, coaches and speedway activists rationalise their training and achieve the highest sport level [4]. Kusznir [7] developed the ‘Speed Your Way’ mobile application dedicated for supporting speedway rider’s training by performing measurements upon their performance on the track. This considered the riders’ speed and tilt towards the motorcycle based on GPS standard and accelerometer and magnetometer sensors. While this concept ensures application’s usability, it does not necessarily provide the most accurate measurements. Most smartphones’ GPS sensors are limited to 1–2 Hz update frequency. However, when it comes to high velocities of even over 100 km/h on the track, receiving location updates every one second was not satisfying. In subsequent work, Martin et al. [8] analysed the physical profile of experienced and inexperienced riders based on anthropometric measurements, including limb length and functional tests. They concluded that low height and weight and a high level of dynamic balance are the key physical features of a high-performance rider [8]. In turn, a recent study by Michalik et al. [1] focused on the comparison of sports results, body composition, the level of anaerobic fitness, and acute cardiorespiratory responses in a maximal anaerobic effort between junior and senior motorcycle speedway riders. It was found that senior riders were characterised by a 4% higher body mass index (BMI) and the mass of adipose tissue by 20.5%. Moreover, body height correlated with all the indicators of the results of senior riders (r = -0.41 to -0.55). This is crucial because a rider with lighter and smaller body size can achieve greater acceleration and maximum speed [9]. It is known that the lower total weight of a competitor and equipment (the minimum weight of a motorcycle is 77 kg) reduces the moment of inertia and inertial load, which, when generating the same power level, has a positive effect on the acceleration phase [8].

In competitive sports, the final success is very often determined by minimal differences or details that occur during sports competitions. An example is the perceptual abilities, i.e. an athlete’s ability to react quickly and the ability to predict, which were found to be beneficial to an athlete’s success [10, 11]. Reaction time (RT) was defined as the time between detecting a sensory stimulus and the behavioural response following it [12]. RT is considered a predictor of the cognitive system’s ability to process information [13]. Previous research determined importance of RT in various sport disciplines [14, 15], compared sex differences between athletes [16] and studied influence of training interventions on reduce RT [17] eg. auditory stimulus training [18]. Therefore, this area should also be explored by researchers involved in motorcycle speedway sports. The importance of reaction time has been considered advantageous in many endurance-based motorsports, i.e. karting, touring car racing and sports car racing [19]. However, to our knowledge, no studies have been published that would determine the importance of a motorcycle speedway rider’s reaction time at the beginning of a heat during the match.

It seems that, as in other racing sports, also in motorcycle speedway riders may benefit from the fastest possible reaction time to impact upon the final results. The capability to achieve a fast RT helps find the right track position for a rider when approaching the first corner. On the other hand, being in the first corner and being in front of the other athletes allows the rider to move freely along the entire width of the track and choose the optimal driving line, increasing the chance of winning the heat [4]. It is critical that a rider obtains a legal and optimal start once the tapes have lifted. According to the motorcycle speedway competition regulations (article 71), a rider who, after the judge has turned the green light on and before lifting the starting machine, moves his motorcycle forwards receives a warning penalty (two warnings will result in exclusion from the heat). However, when a rider touches or tears off the starting tape by motorcycle, he is immediately excluded from the heat [20]. Unfortunately, the regulations above regarding the start of a motorcycle speedway race provoked competitors to make banned movements. In order to react as quickly as possible, irregularities occurred most often, i.e. simple body movements with the motorcycle caused by operating the clutch lever. Due to the problems of judges with recognising the unacceptable behaviour of motorcycle speedway riders at the start and the arising disputes, from the season of 2021, a telemetry system was introduced to the Polish speedway competiion league PGE Speedway Ekstraliga. The system, aimed to objectify the start procedure, assesses the reaction time and controls the correct operation of the starting machine. The start of the heat is strictly controlled to ensure the fairness of the competition. The referee record athletes’ response times in competition to ensure that no athletes gain an unfair advantage by responding in < 0.10–0.12 s after the start signal (false start limit).

Analyses regarding the start phase of elite motorcycle speedway riders allow an understanding of their impact on the rider’s outcomes. Therefore, this work aimed to determine whether: 1) the reaction time to the starting signal affects the points scored by riders, 2) it depends on the starting position (gate) and track geometry, 3) it differs between junior and senior motorcycle speedway riders, 4) it changes during the competitive season. The following hypotheses were put forward: i) scoring points by riders is negatively correlated with RT; ii) a given starting position (gate) and track width determine RT; iii) RT will be faster in the case of senior riders compared to junior riders; iv) the riders will have faster reaction times in the knockout phase.

## Material and methods

### Subjects

The study presents a database of simple reaction times to the starting signal from 65 elite male riders (age 25.9±7.6, min 16.0, max 46.0 years) who competed in the Polish speedway competition league (PGE Speedway Ekstraliga). They represented eight different teams. The maximum age of a rider is not subject to any restrictions, however they must be at least 16 years old to start competing in a league match, having previously passed the motorcycle speedway license exam. Athletes not complying with the inclusion criteria were rejected from the experiment. Parental and participant written informed consent was obtained prior to the investigation. This study was approved by Wroclaw University of Health and Sport Sciences Research Ethics Committee (16/2019) and conducted in accordance with the Declaration of Helsinki.

### Procedures

The matches in which telemetry measurements were used took place between April and September 2021, during the motorcycle speedway competitive season in Poland. The starting reaction times in the main round were measured in each of the 14 rounds of PGE Ekstraliga during one selected match (out of 4 matches in a given round)–a total of 14 matches. In the knockout phase, measurements were taken for each match: the semifinal (4 matches), the bronze medal (2 matches) and the final (2 matches)–a total of 8 matches. In total, measurements were taken during 22 matches. One hour before the first heat, the riders performed an individual warm-up following the recommendations of the club’s coaches.

The classification lists of players from the 2021 competitive season published in the public domain of the organiser of the Polish speedway competition league (PGE Speedway Ekstraliga) were used to determine:

- the starting position (gates from A—closest to the inside part of the track to D—closest to the outside part of the track),
- points scored in the heat (from 0 to 3),
- heat number (from 1st to 15th),
- match result (win, loss, team draw),
- the phase of the competitive season, including rematch matches (main), semifinals and finals (knockout),
- age category (junior riders up to 21 years of age and senior ones from 22 years of age and more),
- the width of the starting straight line of the track (10–12 meters).

### Measuring reaction time

The dataset from the telemetry system was made available to the public in the mobile application PGE Ekstraliga ver. 1.0.66 (PGE Ekstraliga, Warsaw, Poland) [21]. In total, the study was based on a collected numerical series consisting of 1261 data records with the values of the racers’ reaction times to the starting signal given by the referee at the start of a heat. The recorded reaction time (RT) was used, which was the sum of the simple reaction time (SRT) to a visual stimulus (lifting the tape of the starting machine) and the motor response (movement of the speedway motorcycle) until the starting line was crossed.

**Fig 1:**
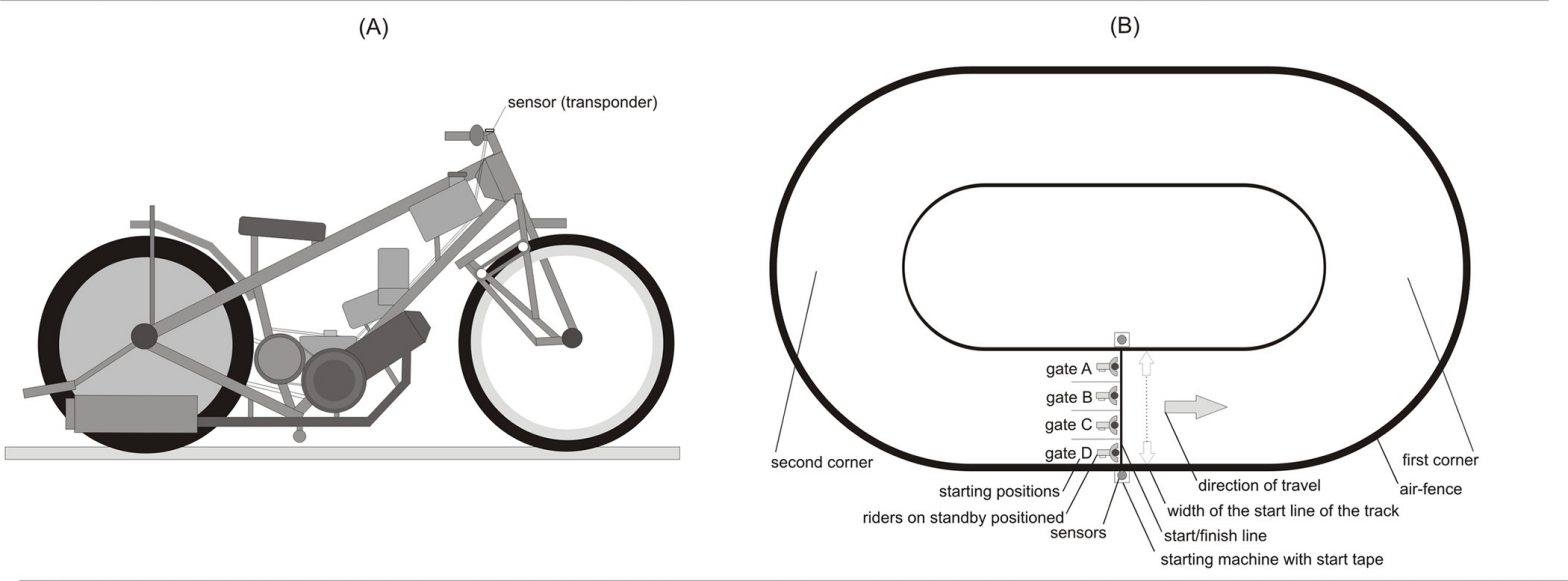
(A) Sensor (transponder) of the Pegasus Speedway © telemetry system (Black Burst, Warsaw, PL) mounted on the handlebars of a speedway motorcycle. (B) Riders on standby positioned in the starting area (gates A, B, C, D) in front of the starting machine with sensors of the Pegasus Speedway © telemetry system (Black Burst, Warsaw, PL).

The computerised Pegasus Speedway © telemetry system (Black Burst, Warsaw, PL) [22] was used to measure RT. The measurement system is designed for speedway competitions, allowing for comprehensive real-time analysis of many geometric/kinematic aspects of a motorcycle speedway rider’s movement on a motorcycle (including heat duration, lap time, the travelled distance, speed, and start reaction time). The system consists of several elements and includes a transponder with a processor and an antenna enclosed in a plastic housing, which is mounted on the handlebars of each speedway motorcycle competing in a match (Fig 1A), two sensors located at the sliders of both poles of the starting machine (Fig 1B), a photo-finish (resolution 1:10000) at the start/finish line to verify the order of competitors at the finish line, an antenna network in the center of the stadium, control software controlled by two network operators.

The starting machine consisted of two poles (internal and external) with tape stretched between them. Each pole was equipped with two electromagnets activating the ratchet mechanism that released the start tape. Using two sliders, the tape was simultaneously lifted up (a starting signal for competitors). The machine was connected to the control panel used by the main judge who started each heat. RT was measured under highly standardised conditions from the moment the start tape was lifted to the first forward movement of the speedway motorcycle, which was recorded with an accuracy of one-hundredth of a second (0.01).

The system relies on wireless GPS data transmission and the triangulation method used to correct the data. The accuracy of the measurement of the spatial identification of the transponder is 14 mm. The real-time data rate is 25 Hz/second in the 2.4 GHz band. In order to establish the relationship between the values indicated by the device and the reference values, the system was calibrated 30 minutes before each match. Before being put into use in PGE Speedway Ekstraliga, the system was positively validated by the manufacturer to obtain repeatable and accurate measurements.

### Statistical analyses

To explore the data, the distribution of the normality was tested using the Shapiro-Wilk test, and the homogeneity of variance was assessed with Levene’s test for all variables in this study. The results were presented in the form of a mean and 95% confidence intervals (CI). In the case of the criterion in which speedway riders were divided into two groups (age category, phase of the competitive season), the statistical analysis used the Student’s t-distribution for independent samples. To determine the practical implications, the effect size was calculated as Cohen’s d according to the following criteria: 0.1—trivial, 0.2—small, 0.5—medium, 0.8—large [23]. To compare the initial reaction time within one criterion, where there were three or more groups, a one-way analysis of variance (ANOVA) was used. A two-way analysis of variance (ANOVA) was performed to determine the influence of selected factors on the reaction time and interactions between them (starting position, points, age category). When a significant F ratio was obtained, a Bonferroni post hoc correction was performed. The effect size was calculated as partial eta-square (η^2^) (small = 0.01, moderate = 0.13, large = 0.26). The p<0.05 level was considered statistically significant.

## Results

This section compares the simple response time with the division into selected categories.

### Starting position

**Fig 2:**
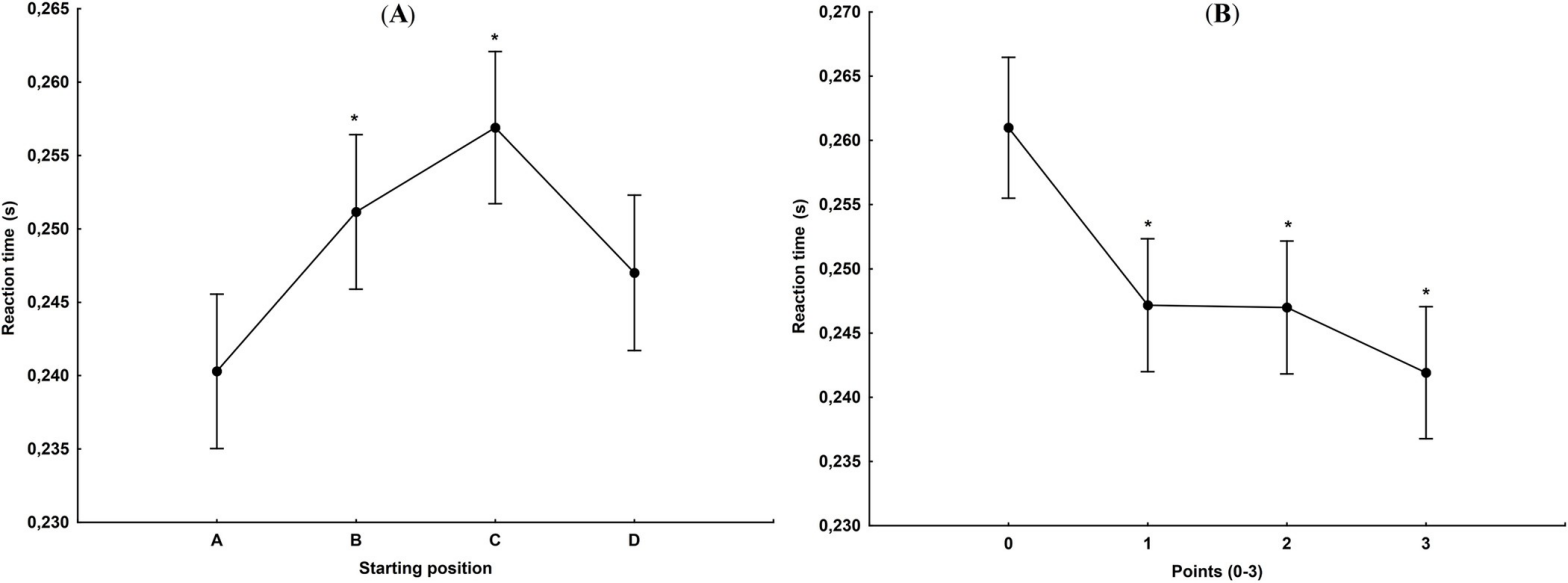
(A) Reaction time in relation to the occupied starting position. *—statistically significant difference at the p<0.05 level in the case of position A. (B) Reaction time in relation to points scored during a heat (in all twenty-two recorded matches of the main and knockout rounds). *—statistically significant difference at the p<0.05 level in the case of 0 points.

The start reaction time differed significantly depending on the occupied starting position F_(3,1257)_ = 6.89, p<0.001, η^2^ = 0.02. In comparison with position A, the average reaction time obtained from the starting positions B (p<0.05) and C (p<0.001) was significantly higher in statistical terms, and for the starting position D (p<0.47) was higher however not significantly (Fig 2A).

### Points earned during a heat

A statistically significant difference was identified in the reaction time compared to the points scored during a heat (F_(3,1257)_ = 8.90, p<0.001, η^2^ = 0.02). Post hoc analysis showed significant differences between 0 and 1 point (p<0.01), 0 and 2 points (p<0.01) as well as 0 and 3 points (p<0.001) (Fig 2B). Reaction time did not affect the final result of a match: winning, drawing or losing (F_(2, 1258)_ = 2.04, p<0.130, η^2^ = 0.003).

### Heat number

**Fig 3:**
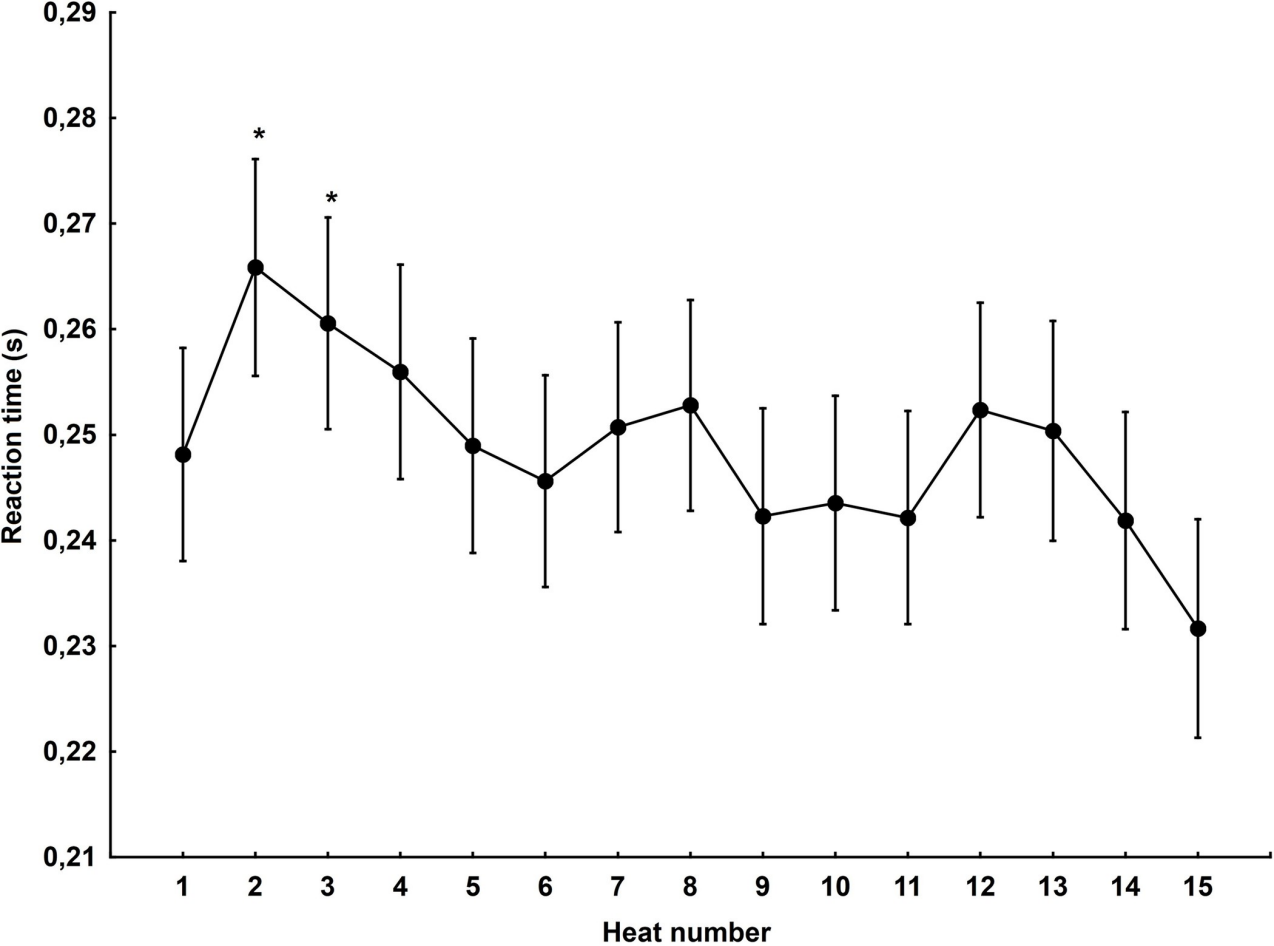
Reaction time in subsequent heats of a motorcycle speedway match. *—statistically significant difference at the p<0.05 level in the case of the 15th heat.

Statistically significant differences were found for the main effect related to the heat number (F_(14,1246)_ = 2.58, p<0.001, η2 = 0.03). Post hoc analysis showed significant differences between heat 15, where the reaction time was the fastest, and heat 2 (p<0.001) and heat 3 (p<0.01) (Fig 3).

### The main phase (a match and a rematch meeting) and knockout phase (a semifinal and a final)

**Fig 4:**
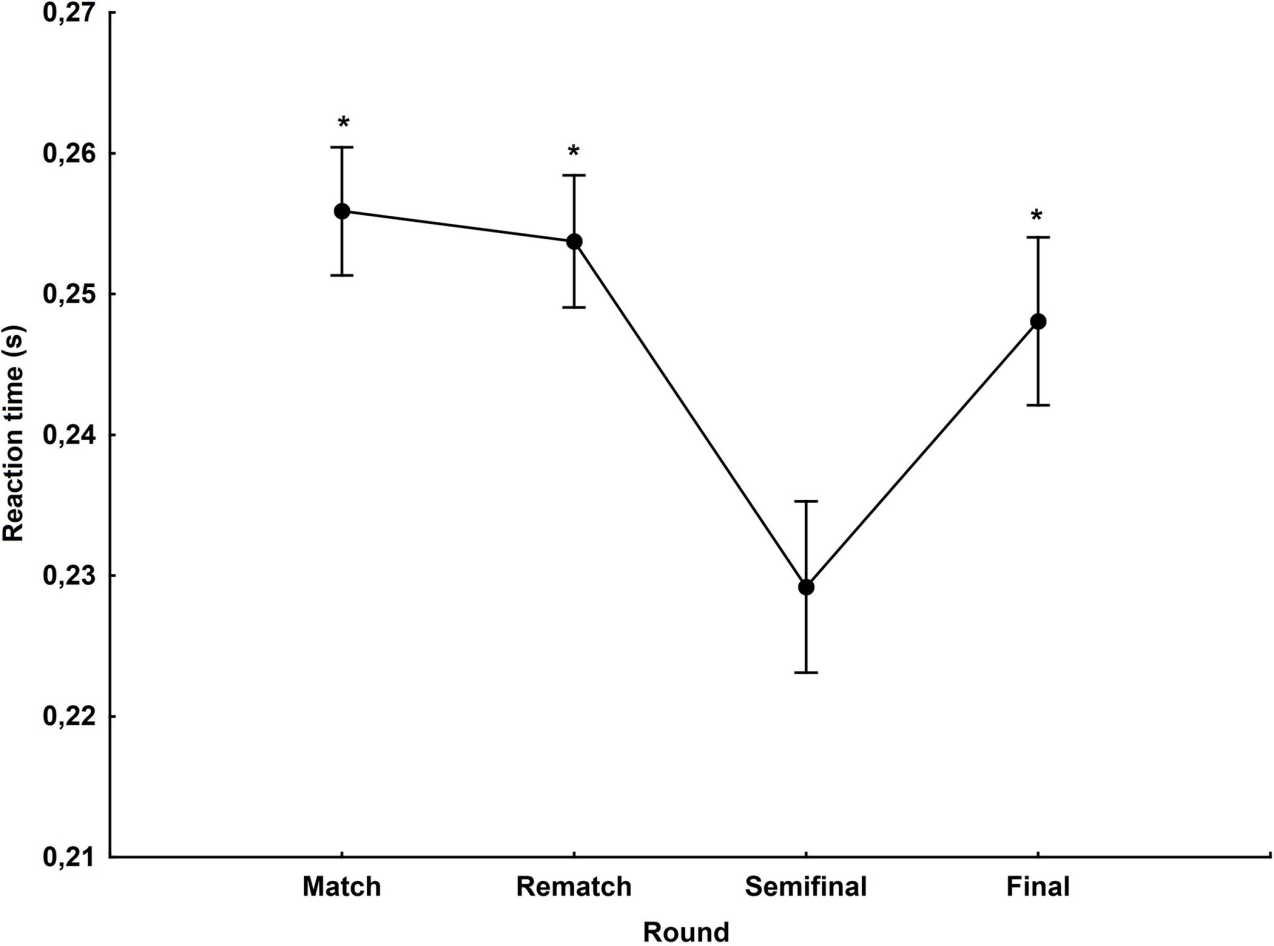
Reaction time during the first match (n = 409), a rematch race (n = 385), a semi-final (n = 229) and the final (a match for gold and bronze) (n = 238). *—statistically significant difference at the p<0.05 level in the case of a semifinal.

The division of the season into the main phase (n = 794) and the knockout phase (n = 467) differentiates the reaction time of riders. The mean start reaction time in the knockout phase (0.239±0.046s) was significantly faster than in the main phase (0.255±0.048s) (p<0.001, d = 0.34). A main effect was identified for round (F_(3,1257)_ = 17.89, p<0.001, η^2^ = 0.04). Post hoc analysis found that in the semifinals, the motorcycle speedway riders achieved the best reaction times (0.229±0.044s) compared to the first match, a rematch race and the finals (all p<0.001) (Fig 4).

### Age category

The mean time of the starting reaction of senior riders (n = 991) was significantly faster than that of junior riders (n = 270) (p<0.001, d = 0.24) and amounted to 0.246±0.05s and 0.258±0.05s respectively in the case of senior and junior motorcycle speedway riders.

### The width of the start line of the track

**Fig 5:**
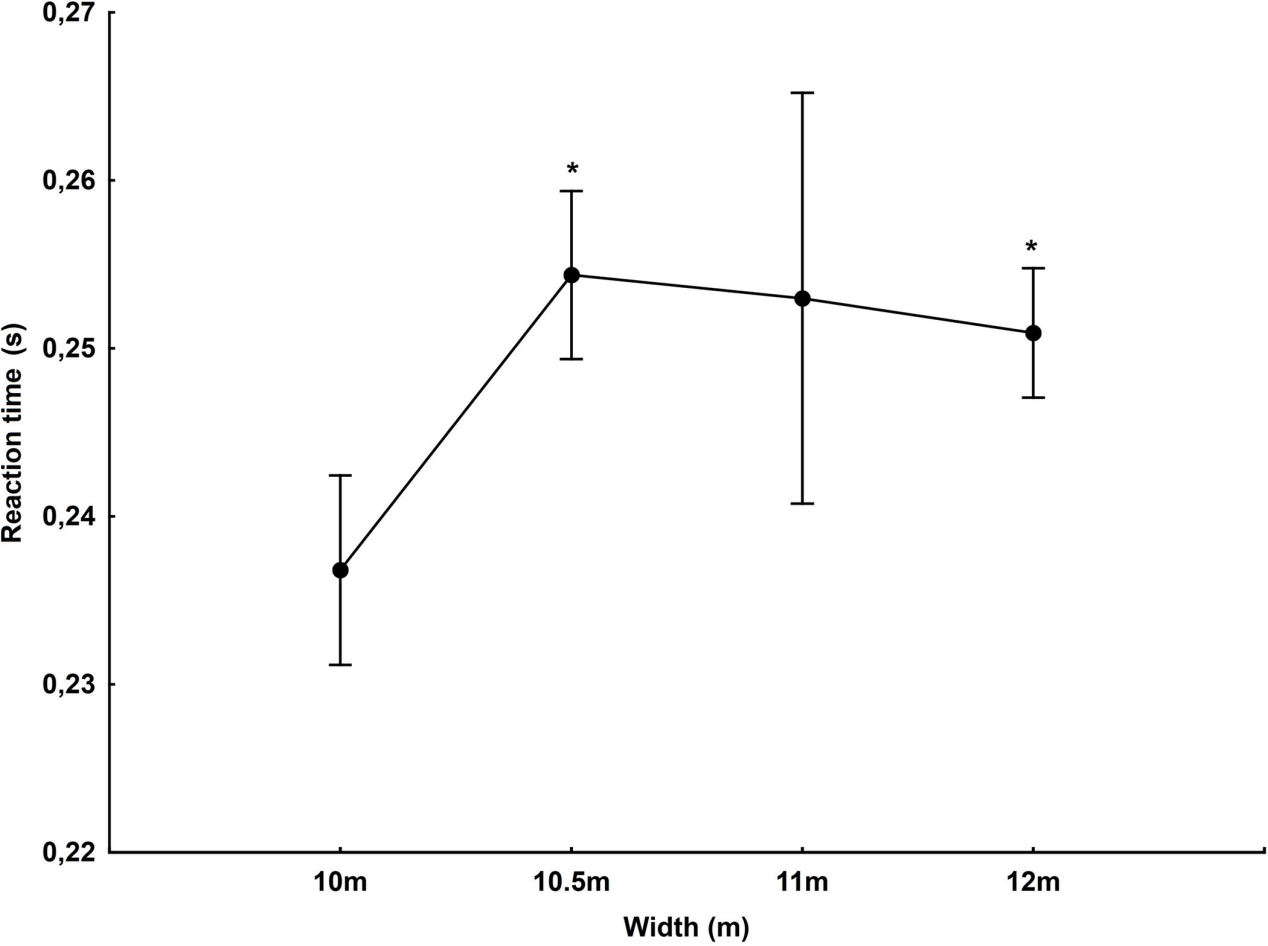
Reaction time depending on the width of the starting line of the track. *—statistically significant difference at the p<0.05 level compared to the 10m track width.

The width of the start line affects the start reaction time (F_(3,1257)_ = 7.94, p<0.001, η^2^ = 0.02). On the track with a width of 10.5 m (p<0.001) and 12 m (p<0.001), the reaction time was significantly slower compared to the width of 10 m (Fig 5).

### Points and starting positions

**Fig 6:**
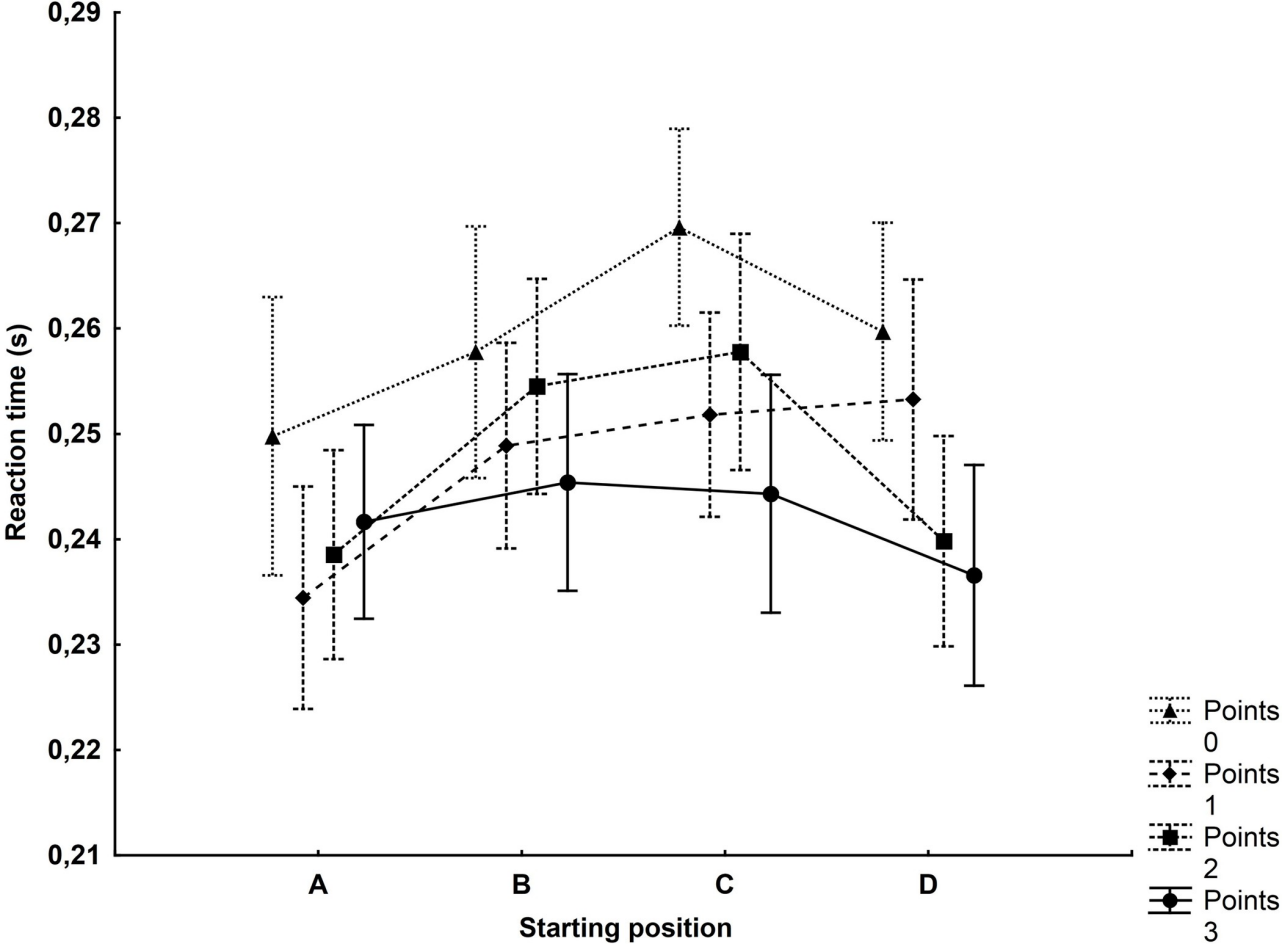
Reaction time depending on the starting position and points scored during a match.

The two-factor analysis of variance showed the main effect for points (F_(3,1245)_ = 6.77, p<0.001, η^2^ = 0.02) and the starting position (F_(3,1245)_ = 5.40, p<0.01, η^2^ = 0.01). There were no significant interactions between the factors (F_(9,1245)_ = 1.11, p = 0.35). Post hoc analysis showed no significant differences in reaction time between the scores for a given starting position. Still, the closest to the assumed significance level (p = 0.09) was the comparison of the reaction time for 0 (0.27s) and 3 points (0.244s) on starting position C (Fig 6).

### The starting position and age category

**Fig 7:**
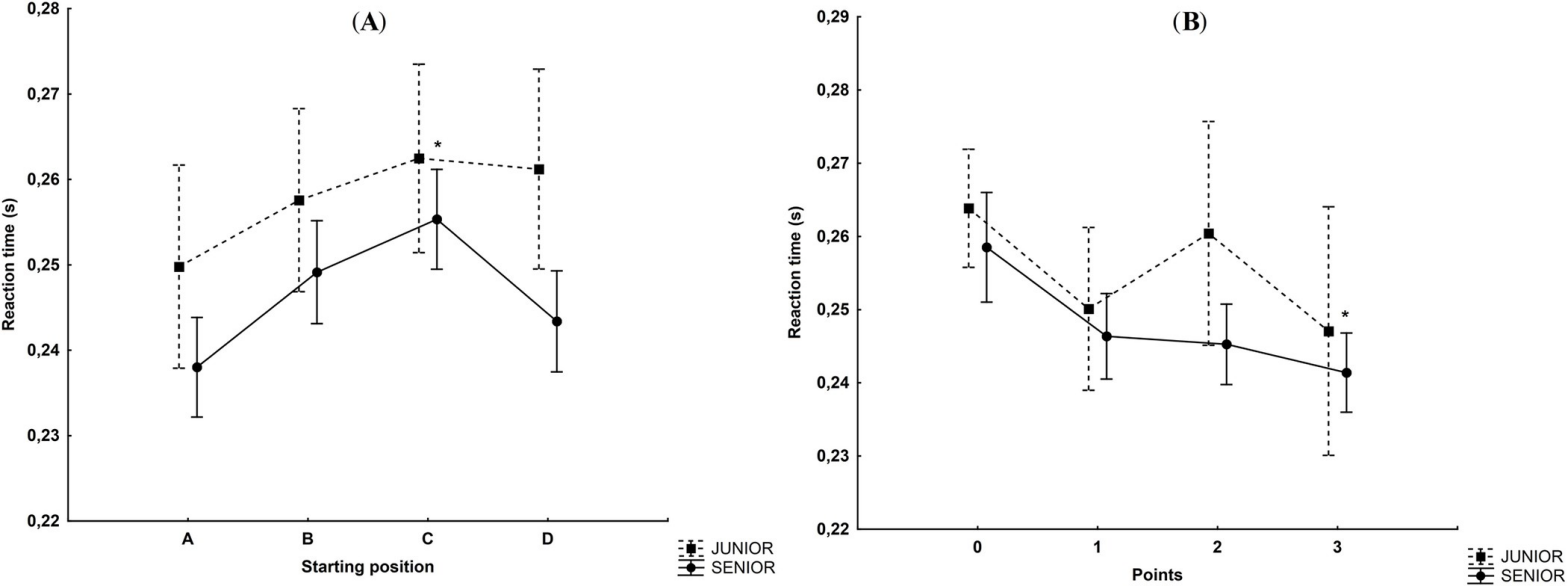
(A) Reaction time depending on the starting positions of senior and junior motorcycle speedway riders. *statistically significant difference at p<0.05 in the case of position A in senior riders. (B) Reaction time depending on the points scored by senior and junior motorcycle speedway riders. *—statistically significant difference at the p<0.05 level in senior riders while scoring 0 points.

The main effect was found for the starting position factor (F_(3,1253)_ = 3.54, p<0.05, η^2^ = 0.008) and the age category factor (F_(1,1253)_ = 12.00, p<0.001, η^2^ = 0.009). Again, there was no effect between the factors (F_(3,1253)_ = 0.53, p = 0.66). The reaction time was significantly worse in senior riders (p<0.001) when they started from position C (0.255s) compared to position A (0.238s) (Fig 7A).

### Points and age category

There was a main effect for points (F_(3,1253)_ = 4.80, p<0.01, η^2^ = 0.01) and category (F_(1,1253)_ = 4.00, p<0.05, η^2^ = 0.003), but no interaction between the factors (F_(3,1253)_ = 0.44, p = 0.72). The reaction time in senior riders was significantly faster (p<0.01) when they scored 3 points (0.241s) compared to no points (0.258s) (Fig 7B).

## Discussion

The data presented in this paper are the first ones to verify the impact of the reaction time, achieved by elite male motorcycle speedway riders on their sports outcomes. Rapid response to a stimulus is a constant requirement for motorcycle speedway riders to start each heat. The better the reaction time during the start, the faster the competitor will move and take a more advantageous position, which can mean an advantage on the first corner where all four riders come closer together while remaining in close contact. Our hypotheses were supported by the results of the study.

When verifying the first hypothesis, it was indicated that the shorter the reaction time, the more points a rider scores. Interestingly, it was also proved that RT had no influence on the final result of a match (p<0.130). These results correspond to the studies by Doggart and Martin [5], Doggart et al. [2] and Martin et al. [4], who studied the relationship between the start and first corner (r = 0.64), starting and finish positions (r = 0.24) and first corner and finish positions (r = 0.42). The findings mean that the starting position (gate) correlates highly with the position on the first corner but is not as well related to the heat result. The authors also state that the result of a meeting is influenced by other factors, such as the level of the players in the team, the mechanical settings of the motorcycle, the track surface, and weather conditions during a match.

Similarly, the second hypothesis was also confirmed. It was found that the occupied starting position significantly influenced RT. Indeed, in terms of RT, the best was gate A, and the worst positions B and C. Based on our results, we consider that it may be more difficult to observe the starting machine sliders, which may help riders get a faster reaction from the start, from the middle positions. An interesting fact was shown by two-factor comparisons (points and starting position). Although there were no significant differences, the slow reaction time (0.270s) indicated 0 points, and the fast RT (0.244s) 3 points in the gate C. On the other hand, when it comes to track geometry, the lowest reaction times were obtained on the narrowest tracks with a straight width of 10 m. It seems, therefore, that the wider the track, the greater the useful field of view of a motorcycle speedway rider [24], which in turn reduces the precise perception of important objects and focuses attention on an essential element of the environment [25], i.e. the start tape. On the other hand, a narrower track implies a better reaction time due to the smaller available space on the first bend. Doggart et al. [2] studied the influence of starting positions on the points scored by motorcycle speedway riders. An analysis of the first gate showed that 23% of starts from that spot resulted in taking the first place in the heat; 68% of starts ended with the first position on the first corner; 53% of starts led to the first place in the first corner and a heat victory. In turn, in the case of the second position, 46% of starts resulted in the first place in the heat; 23% of starts led to an advantage over rivals on the first corner; 60% of starts were related to reaching the first position on the first corner and winning a heat at the same time. In addition, Williamson [6] carried out a statistical analysis of the starting numbers (identical to the starting positions) awarded to motorcycle speedway riders during the world championship cycle (Speedway GP). In the conclusions, the author stated that the starting positions drawn by the riders predispose them to obtain specific results. Therefore, suggesting changes in the competition regulations.

Thirdly, it was assumed that there could be differences in RT values due to riders’ experience. Senior motorcycle speedway riders (0.246s) showed significantly faster RT values compared to junior ones (0.258s). Additionally, more detailed two-factor comparisons in terms of points scored in the age category showed that RT in senior riders was significantly lower (p<0.01) when they scored 3 points (0.241s) compared to no points (0.258s). However, taking into account the occupied starting position and the age category of senior riders, RT was significantly slower when they started from gate C (0.255s) compared to gate A (0.238s). These results indicate that speedway experience is positively associated with a faster RT. This is consistent with many reports in which it was reported that in individual and team disciplines, competitors with greater training experience presented shorter reaction times [26]. Experienced athletes make decisions faster than beginners, which can be explained by both training and routine gained during competitions [27]. This is also confirmed by Milic et al. [28], in which experienced fencers had a faster reaction time than beginners. Moreover, the most significant differences between experienced and novice athletes in terms of reaction time were observed in disciplines where coordination between a competitor’s body parts and the held utensil is required, such as American football or basketball [29]. Similarly, in motorcycle speedway racing, motor coordination is vital [8]. It is worth noting that a learning effect is also possible as a result of starts (skill) [30]. Therefore, the starting point of an experienced competitor is more effective than that of a beginner. In addition, a highly qualified athlete anticipates action and then processes information in advance so that the traffic organisation system does not have to react to unforeseen events [31]. In addition, seasoned competitors have the ability to use perceptual cues effectively. Hence, they are able to ignore a large part of the signals (which may distract young riders) by focusing on the crucial stimuli for the effective implementation of a motor task [27]. Elite athletes react quicker than less skilled athletes, however, at present the evidence for this is incoherent. Eikenberry et al. [32] did not find a difference in RT between experienced and novice track and field sprinters. Similarly, international-level runners had a slower block RT compared with national-level athletes [33]. We consider that in order to explain the indicated differences between these groups of competitors, it is justified to conduct standardised laboratory tests of reaction time as well as kinesthetic sensation, which were performed on selected representatives of motorsports [34].

Finally, significant differences were shown during the subsequent heats in a match and the phases of the competitive season. Between heat 15, which has the shortest reaction time, and heats 2 and 3, all riders are under constant pressure which forces them to shorten their decision-making time and simple reaction time. This is especially the case in the 15th heat, where the best riders in terms of scored points compete. Contrary to heat number 2, to which only junior motorcycle speedway riders are selected, it is characterised by a worse reaction time, which was shown earlier. The RT difference between heat 2 and 15 could be also due to riders being more likely to adopt risk during the end of the match. That is in line with some studies showing that in track and field RT was faster on average during the final round of competitions compared with the first-round heats. It emphasizes perhaps that the faster sprinters who took part in the final rounds had better RT than slower sprinters eliminated in first-round heats [35, 36]. It suggests that residual fatigue may not exist during the match, but further research are needed to explain this observation. On the other hand, a detailed analysis of the two parts of the motorcycle speedway competitive season showed that the average RT in the knockout phase (0.239s) was significantly shorter than in the main phase (0.255s). Moreover, in the semifinals, the riders achieved the best reaction times (0.229s) compared to the first match of the competitive season, the rematch meeting and the finals. Likely, the riders’ internal and external expectations related to the inaugural and final match may have delayed their response time. This is also consistent with the results of studies in which drivers under pressure responded to stimuli later compared to conditions without pressure [37]. The pressure to win and the awareness of failure are essential sources of emotional arousal in the central nervous system. According to the inverted U theory, a high level of arousal is only effective to a certain extent, but increasing it further actually worsens reaction time [38]. Additionally, high arousal levels appear to interfere with muscle control and decision-making [39].

Several potential limitations must be considered when designing future studies of this type. First, the study sample consisted only of elite motorcycle speedway riders (junior and senior ones), therefore the results cannot be generalized in the context of lower competition league. Secondly, the reaction time depends on many variables such as somatic (age), environmental (track width), or motivational factors (pre-start fever, tension before the decisive heat). Furthermore, it should be noted other factors such as crowd pressure, and financial gratification (paid bonuses for gained points by riders), may also impact RT. It would be interesting to know the effects of drinking energy drinks by athletes given various brands heavily sponsors the sport, however, is beyond the scope of this study. Future tests of motorcycle speedway riders should be performed under standardized laboratory conditions, which would allow for a more detailed understanding of reaction time determinants in motorcycle speedway. Contrary to limitations, one of the strengths of this study is that RT was assessed in realistic situations during matches.

This study provides some important practical applications on the conditions of the starting reaction time in motorcycle speedway racing. Hence, the presented results can help to create specific training formulas used to improve reaction time among young and experienced motorcycle speedway riders. Motorcycle speedway riders are required to continuously visually predict where the start tape is going to be during the starting moment. We propose introducing regular exercises to improve the reaction time in the training of riders. It is reasonable to use mobile starting machines with a non-standard, narrower construction spacing. Since a more visible stimulus increases the speed of decision-making [40], observing the sliders may result in a shorter reaction time under given conditions. We also recommend that coaches pay special attention to the quality of the start from individual starting positions, especially from gate C, where the average RT was significantly the worst. Finally, we suggest that to maintain the objectivity of the starting phase, these parts of the machine should be carefully concealed from competitors so that only the starting tape is the stimulus. Admittedly, riders change their starting positions after each heat. Nevertheless, they can benefit from observing the sliders every time. This problem should be solved in the future by studying eye movement using advanced eye-tracking technology. The indicated application values bring new practical knowledge to motorcycle speedway racing.

## Conclusions

Findings from this study provide multiple points of application to motorcycle speedway riders’ training. It was confirmed that the fast straight response time to the starting signal allows motorcycle speedway riders to score more points in the heat but does not determine the final result of a match. In terms of RT, the best was gate A, and the worst positions were B and C. Regarding track geometry, the lowest reaction times were obtained on the narrowest tracks. In addition, senior motorcycle speedway riders disclosed significantly faster RT values than junior ones. Moreover, in the 15th heat, where the best riders compete in terms of scored points scored in the match, the shortest reaction time was recorded. By contrast, the 2nd heat, where only junior riders are designated, is characterized by a significantly worse reaction time. Finally, the analysis of the two parts of the motorcycle speedway competitive season showed that the average RT in the knockout phase was shorter than in the main phase.

To summarize, the study indicates that in training, riders can pay attention to the starting positions (gates), the width of the starting straight, and riders’ experience during practice, and use various training variants of these factors. In general, we suggest that improving the simple reaction time to the starting signal should become one of the important training goals of motorcycle speedway riders, also taking into account disruptive factors that occur during an actual competition.

This study contributes to the level of knowledge available in the literature about motorcycle speedway sport, but some limitations should be considered. Future work should aim to establish any other factors such as neuro-physiological and non-neuro-physiological components that could affect RT among motorcycle speedway riders.
